# 

**DOI:** 10.1192/bjb.2018.44

**Published:** 2018-12

**Authors:** Martin Curtice

**Affiliations:** Consultant in Old Age Psychiatry, Worcestershire Health and Care NHS Trust, New Haven, Princess of Wales Community Hospital, Bromsgrove, UK; email: mjrc68@doctors.org.uk

This book is the fruition of an idea by the author that has germinated since the 1970s. It was largely inspired by his concern with unfair discrimination against people with mental illness. From its evocative title to its thought-provoking review of 200 years of mental health legislation (both in the UK and globally), this is a book that should appeal to a wide audience. It has been deliberately written to include patients and family (‘experts by experience’) as part of a multidisciplinary audience. The fluent and conversational writing style, the purposeful use of ‘typical’ case histories, the text being well signposted with subheadings, and references being readily accessible at the end of each chapter certainly help in this respect.

There are two main themes – the use of coercion in mental health practice and an equally interesting bold and well-thought-out exposition of a proposed new mental health legislation termed a ‘Fusion law’. This law is non-discriminatory and generic for all specialties, with no need for specific mental health law, the framework being based on decision-making capability and a modified best interests approach to involuntary admissions and treatment decisions.

The book has five parts and comprises 13 chapters. Part I considers problems associated with involuntary detention, which the author believes actually contributes towards discrimination against people with mental illness. Part II offers an exciting solution to involuntary detention and treatment and proposes in-depth new legislation. This part has a more theoretical or academic feel, but this is where the author elucidates and validates his proposed reforms. A visual flowchart of these reforms may have been helpful to help understand these better. The author does acknowledge that there would need to be some initial interim measures in the forensic setting were these reforms enacted. Part III analyses coercion and treatment pressures in everyday clinical practice and provides ideas to try and reduce recourse to coercive measures in both detaining and treating people. This part would be particularly useful for junior doctors of all specialties. Parts IV and V are about translating the theoretical basis of new reforms into the reality of clinical practice across all physical and mental health settings.

Given that mental health law has not fundamentally changed for around 200 years (essentially still being based on the two criteria of having a mental disorder and posing a risk of harm, but *not* including any form of capacity criteria) it would surely be an epoch-making quantum leap in mental health legislation to enact this Fusion law. However, the author opines that ‘change is now essential’ and is optimistic that such legislation favouring patient empowerment will continue to emerge globally, citing a version of Fusion law and its principles having been passed in the Northern Ireland Mental Capacity Bill 2016. This was an invigorating read, challenging the long-held orthodoxy of mental health law. It is a text that will surely resonate with all clinicians involved in the application of the Mental Health Act.

